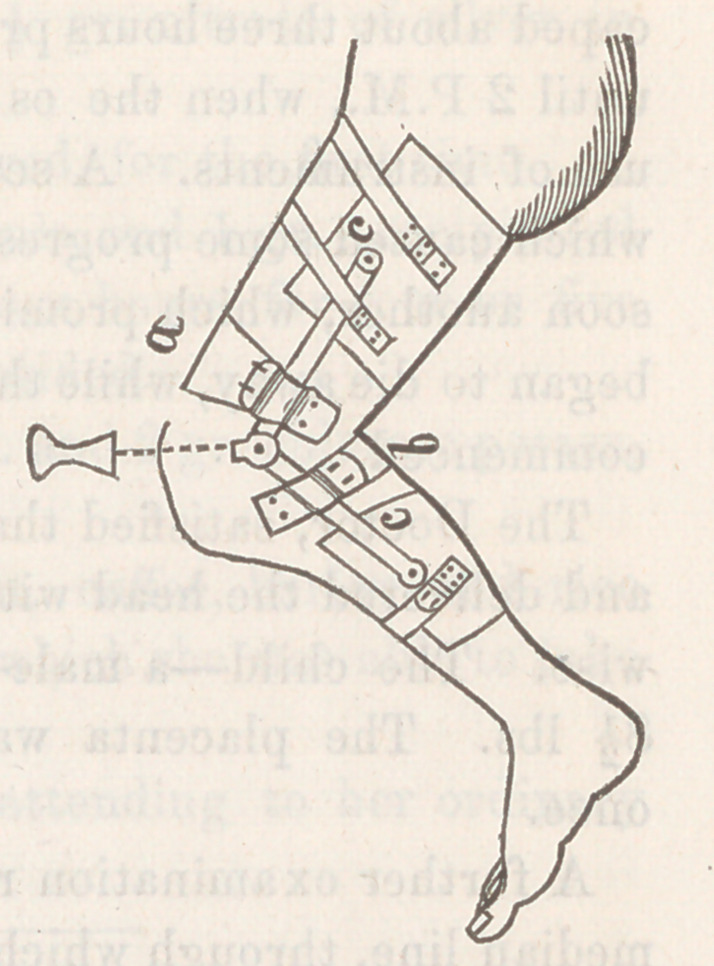# Anchylosis of the Knee

**Published:** 1868-10

**Authors:** J. S. Sherman

**Affiliations:** Chicago


ARTICLE XXXIX.

### ANCHYLOSIS OF THE KNEE.

By J. S. SHERMAN, M.D., Chicago.

Of all deformities resulting from diseases of joints, none yield quicker, or show better results from treatment, than those of the knee. Its position is such that mechanical force can be used to great advantage.

Yet failures to completely straighten these limbs are not uncommon results, and they are generally due to two causes: First, the force used to extend them is not sufficient; second, the relative position of the bones is not recognized. If we flex the knee, the tibia is carried backward upon the posterior portion of the articulating surface of the femur, and in order to extend it, and restore its position to a straight line, we must not only extend, but bring the head of the bone forward. It is from failure to accomplish this that deformity of the knee remains after the foot has been brought to the ground; and the joint presents the appearance of a partial dislocation backwards.

The ligaments at the posterior part of the joint are not sufficiently elongated to allow the tibia to resume its natural position upon the femur. The latter, therefore, with the patella, project beyond the spine of the tibia.

If the anchylosis is of a character justifying immediate extension, the limb may be brought down by forcibly rupturing the ligaments. If the slow method is preferred, the apparatus figured will be found most satisfactory in its action. It is capable of all the power produced by the old method of extension by means of a rod and screw. It distributes the pressure evenly upon the thigh and calf, and with it the limb can be carried beyond a straight line, which is often absolutely necessary to prevent future contraction. Its force has a double bearing—bringing the tibia forward as well as extending it.

The construction is simple, and, applied as represented in the cut, its action is efficient. Two pieces of sheet-iron, bent to fit the thigh and calf, are connected on the sides by a steel bar, provided with a ratchet opposite the knee-joint, and attached at the points (*c*, *c*) with a *movable* rivet, so that their motion in straightening the limb does not change the position of the sheet-iron plates, but allows them still to keep perfectly in contact with the limb. Two strong elastic bands (*a*, *b*) are buckled to the side-rods; one passing over the femur, and drawing it backward; the other behind the tibia, forcing it forward. Extension is made by means of the ratchet at the knee. The bringing forward of the tibia may be accomplished by putting the patient to bed, enclosing the leg in a plaster bandage, and applying the force by means of a pulley and weight. But the majority of these patients are able to get about with crutches, and confinement to bed should be avoided, if possible.

## Figures and Tables

**Figure f1:**